# Genome Sequence of Metarhizium *rileyi*, a Microbial Control Agent for Lepidoptera

**DOI:** 10.1128/MRA.00897-19

**Published:** 2019-09-05

**Authors:** Eliseu Binneck, Claudia Cristina López Lastra, Daniel R. Sosa-Gómez

**Affiliations:** a Embrapa Soja, Londrina, Paraná, Brazil; b Centro de Estudios Parasitológicos y de Vectores, La Plata, Buenos Aires, Argentina; Vanderbilt University

## Abstract

Metarhizium rileyi (formerly known as Nomuraea rileyi) is a potential agent for microbial control of many insect pests from the order Lepidoptera, the damages of which can cause considerable loss of productivity in agriculture. We report the genome sequence and annotation of M. rileyi strain Cep018-CH2/ARSEF 7053.

## ANNOUNCEMENT

Metarhizium rileyi is a cosmopolitan species of entomopathogenic fungi of the family Clavicipitaceae (Hypocreales, Ascomycota) with extensive literature published under its synonym Nomuraea rileyi (1, 2). The main susceptible species of insects, which are key pests of crops such as cotton and soybean, belong to the lepidopteran families Noctuidae, Erebidae, and Nymphalidae (3–7). Metarhizium rileyi usually presents high genetic variability (8), which has been closely related to the host species from which it is isolated (4, 9, 10). Unlike other most common fungal entomopathogens with the greatest known epizootic potentials, such as Metarhizium anisopliae and Beauveria bassiana, M. rileyi has a narrow spectrum of hosts (10, 11). Because of its high selectivity and effective control under natural or agricultural conditions, *M. rileyi* is an attractive biocontrol agent with potential for development as a bioinsecticide (3, 5) or for prospecting potential biologically active compounds with many possible uses. The genome data of *M. rileyi* strain Cep018-CH2/ARSEF 7053 were obtained, with the aim of providing additional insights into fungal diversity and interactions with the host.

*M. rileyi* strain Cep018-CH2 was isolated from a velvetbean caterpillar (Anticarsia gemmatalis) on 4 April 2001, in Chivilcoy (Buenos Aires Province, Argentina), and it was deposited at the Centro de Estudios Parasitológicos y de Vectores (CEPAVE) collection, La Plata, Buenos Aires, Argentina, and also at ARSEF (12) under the accession number ARSEF 7053. A single-spore culture of Cep018-CH2/ARSEF 7053 was prepared on Sabouraud maltose agar with yeast extract (SMAY; 2.5 g of neopeptone, 10 g of maltose, 2.5 g of yeast extract, 3.75 g of agar, and 250 ml of water) at 26°C for 5 to 7 days. Conidia were inoculated in 50 ml of SMAY broth with shaking at 250 rpm at 26°C for 8 to 10 days. The fungal mycelia were collected by filtration and washed with sterile distilled water, and the DNA was extracted using a modified cetyltrimethylammonium bromide (CTAB) protocol (1). The preparation of libraries and sequencing were performed at Fasteris SA (Plan-les-Ouates, Switzerland). Whole-genome sequencing was carried out on an Illumina HiSeq platform using four libraries, two Fasteris *de novo* (Fasteris SA) paired-end libraries with insert sizes of 250 to 350 bp, and two Nextera (Illumina, Inc.) mate pair libraries with insert sizes of 3,000 and 5,000 bp, producing a total of 182,506,935 read pairs. The average read length was 125 bp for both sets of libraries. The quality of the sequencing raw reads was assessed with FastQC version 0.11.5 (13). Default parameters were used in all analyses, unless otherwise stated.

The reads were quality filtered and assembled into scaffolds using the ALLPATHS-LG pipeline version r52488 (14). The generated assembly was evaluated using QUAST-LG version 5.0.2 (15). The final assembly had a total length of 31,808,756 bp, with 1,044 contigs joined into 249 scaffolds, 240 of which were larger than 1,000 bp. The lengths of the longest and *N*_50_ scaffolds were 2,535,063 bp and 815,204 bp, respectively, and the *L*_50_ value was 10. The overall G+C content was 51.30%.

Gene prediction and annotation using Funannotate version 1.5.0-b99af2c (16), with the gene predictors AUGUSTUS version 3.3.1 (17), GeneMark-ES Suite version 4.35 (18), and tRNAscan-SE version 2.0.0 (19), resulted in 8,945 protein-coding and 102 tRNA genes. This annotation is comparable with that of *M. rileyi* RCEF 4871 (GenBank accession number AZHC00000000), which has a total assembly length of 32,013,981 bp and 8,764 protein-coding genes (20). Secondary metabolite analysis was performed using antiSMASH fungal version 4.2.0 (21), identifying 30 gene clusters involved in the biosynthesis of specialized metabolites, 480 biosynthetic enzymes, and 155 secondary metabolism Clusters of Orthologous Groups (smCOGs). Functional annotation of the predicted proteins, by pattern matching with the Pfam (22), UniProtKB/Swiss-Prot (23), eggNOG (24), CAZy (25), MEROPS (26), InterPro (27), and antiSMASH (28) databases, as well as comparison to other entomopathogenic fungal genomes, revealed key genes coding for peptidases, carbohydrate-active enzymes, secreted proteins, and transcription factors.

### Data availability.

This whole-genome shotgun project has been deposited at DDBJ/ENA/GenBank under the accession number SBHS00000000. The version described in this paper is version SBHS01000000. The raw reads were deposited in the NCBI Sequence Read Archive under the BioProject accession number PRJNA503201.
